# Assessment of the Occlusal Support Area in Children with Mixed Dentition: A Clinical Cross-Sectional Study from Oradea, Romania

**DOI:** 10.3390/children13101323

**Published:** 2026-09-29

**Authors:** Emilia Albinița Cuc, Rahela Tabita Moca, Diana Berechet, Gabriela Ciavoi, Raluca Iurcov, Ioan Andrei Țig, Mihai Benchea, George Daniel Cuc

**Affiliations:** 1 Department of Dentistry, Faculty of Medicine and Pharmacy, University of Oradea, 4 Universității Street, 410087 Oradea, Romania; cucalbinita@uoradea.ro (E.A.C.); moca.rahelatabita@didactic.uoradea.ro (R.T.M.); gciavoi@uoradea.ro (G.C.); riurcov@uoradea.ro (R.I.); itig@uoradea.ro (I.A.Ț.); bencheamihai@yahoo.com (M.B.); 2 Cluj-Napoca Emergency Clinical County Hospital, 3-5 Clinicilor Street, 400347 Cluj-Napoca, Romania; cuc.georgedaniel@gmail.com

**Keywords:** tooth, deciduous, tooth loss, dentition, mixed, dental caries, child, cross-sectional studies, Romania

## Abstract

**Background/Objectives:** The integrity of the primary teeth in the occlusal support area is essential for the eruption of the permanent successor teeth and for the development of a functional occlusion. This study aimed to clinically evaluate the health status of the primary teeth in the occlusal support area among children in the early mixed dentition attending an orthodontic practice in Oradea, Bihor County, Romania, and to analyze the pattern and prevalence of damage to this area by age and sex. **Methods:** This observational, cross-sectional, descriptive-analytical study was conducted at a specialized orthodontic practice in Oradea, Romania, between January and June 2026. A total of 255 patients aged 6–9 years (136 girls, 119 boys) were included, selected through consecutive sampling from an initial group of 308 patients. The evaluated variables included the integrity of the occlusal support area, the presence of caries, premature extractions, their combination, location, and the type of extracted primary molars. Comparisons between age and sex groups were performed using Fisher’s Exact Test, with Bonferroni-corrected Z-tests. **Results:** The occlusal support area was damaged in 72.2% of patients, most frequently through a combination of premature extractions and caries (52.7%). Damage increased significantly with age, from 54.8% at 6 years to 80% at 8 years (*p* = 0.032). The second primary molar was the most frequently extracted tooth (62%). No significant sex-based differences were observed across the overall sample, except for a significant association at 7 years of age (*p* = 0.001), where girls more frequently presented isolated premature extractions. **Conclusions:** The occlusal support area is frequently affected in children with early mixed dentition who attended the orthodontic practice from Oradea, Romania, with damage increasing progressively between 6 and 8 years of age. These findings support the need for active monitoring and early preventive interventions during this critical developmental period.

## 1. Introduction

The integrity of the teeth in the occlusal support area is essential for maintaining the space required for the eruption of the successional permanent teeth and, subsequently, for the formation of a functional occlusion [1]. Following the eruption of the lateral incisors, around the age of 8 years, a region encompassing the canine and the primary molars becomes delimited between these incisors and the first permanent molar, an area referred to by Korkhaus as the “occlusal support area” [2]. This designation reflects the crucial role of these teeth in stabilizing and maintaining interarch relationships during the primary dentition [2].

During the mixed dentition period, following the emergence of the first permanent molar and the lateral incisor, this area plays a determining role in preserving the space required for the eruption of the permanent canine and premolars [3]. The morphological and functional integrity of the primary teeth in this region ensures both adequate space for the permanent successors and their correct eruption, thereby influencing the development of a harmonious occlusion in both the vertical and horizontal planes [4].

Simple or complicated caries, as well as premature extractions of primary teeth, significantly affect this space [5]. Early loss leads to mesial migration of the first permanent molar, causing eruption disturbances of the successional teeth, dental crowding, ectopic eruptions, tooth impactions, and ultimately dysfunctional occlusion, with repercussions on the dentofacial development [4,5].

From a dimensional standpoint, the available arch space results from the balance between the mesio-distal dimensions of the teeth and arch length. Recent studies show that discrepancies between the “space required” (determined by the size of the permanent teeth) and the “space available” (determined by arch length and the position of the primary teeth) represent a critical factor in the occurrence of dental crowding and eruption disturbances during the mixed dentition period [6,7,8,9]. This relationship makes the morphological and positional integrity of the primary molars essential for preventing mesial migration of the permanent first molar. The early loss of primary teeth in this area is consistently associated in the current literature with space loss, dental migration, and the subsequent development of malocclusions [4,5,6,10].

The migration of the neighboring teeth following premature tooth loss leads to a reduction or even complete closure of the space intended for the permanent teeth, affecting the chronology of eruption and occlusal relationships, and potentially generating dental extrusion, collapse of the occlusal plane, or the onset of lateral crossbites [4,5,11]. The consequences are complex, both morphological and functional, and their severity depends on the type and number of teeth affected, the rate of tooth loss, the length of time before the successor erupts, and the biological timing of replacement [5,12].

The early loss of the mandibular second primary molar leads to significant dimensional changes at the arch level, the most notable of which is mesial migration of the permanent first molar, considered the main etiological mechanism underlying space loss and the subsequent occurrence of malocclusions [13]. The mandibular second premolar, lacking sufficient space, may either remain impacted or erupt buccally or lingually, resulting in an apparently mesialized occlusion with multiple interferences [5]. The early loss of the maxillary primary canine may lead to transposition of the lateral incisor or the first premolar, depending on the timing of the loss [14]. In the mandible, loss of the primary canine leads to premature eruption and mesial migration of the permanent canine, reducing the space available for the lateral incisors [15,16].

Given these implications, assessing the health status of the primary teeth in the occlusal support area in pediatric populations becomes essential. In Romania, the increased prevalence of dental caries among children and unequal access to preventive dental services may amplify the risk of damage to these essential structures [17]. Moreover, there is limited data specifically addressing this issue in local populations, including in Bihor County, where socio-economic particularities and the profile of access to care may influence the development of the mixed dentition. In this context, clinical evaluation of the occlusal support area and of the consequences of its impairment represents a necessary step toward understanding local risks and toward substantiating preventive and interceptive interventions aimed at children in this region.

Thus, the aim of this study was to clinically evaluate the health status of the primary teeth in the occlusal support area among children in the early mixed dentition attending an orthodontic practice in Oradea, Bihor County, Romania, as well as to analyze the pattern and prevalence of damage to this area (caries, premature extractions, and their combination) according to age and sex.

## 2. Materials and Methods

### 2.1. Study Design and Patient Group

The present study is an observational, cross-sectional, descriptive-analytical study, conducted at a specialized orthodontic practice in Oradea, Romania, between 1 January 2026, and 30 June 2026. The study was conducted in accordance with the Declaration of Helsinki and was approved by the Research Ethics Subcommittee of the University of Oradea, approval No. 56, dated 22 December 2025 (registered under No. 20855). Written informed consent was obtained from the parents or legal guardians of all children included in the study prior to enrollment.

A consecutive sampling method was used, with all eligible patients presenting at the aforementioned practice for orthodontic treatment during the study period (1 January–30 June 2026) being included in the initial evaluation. No a priori sample size calculation was performed. The sample size was determined by the number of eligible patients presenting during the six-month recruitment period. The initial group consisted of 308 patients in the first phase of mixed dentition, aged between 6 and 9 years (166 girls, 142 boys). The children attended the same practice and originated from the same geographic area (the city of Oradea and the other localities of Bihor County). After applying the eligibility criteria described below, 255 patients were retained for the final analysis.

The inclusion criteria were: age between 6 and 9 years; first phase of mixed dentition; complete orthodontic documentation (panoramic radiograph and study models); and no history of previous orthodontic treatment.

The exclusion criteria were: previous orthodontic treatment; history of dental trauma involving the primary or permanent dentition; congenital absence of teeth or supernumerary teeth; craniofacial anomalies, genetic syndromes, or systemic or developmental disorders affecting the dentition; and incomplete orthodontic documentation (missing panoramic radiograph or study models). Information on medical and dental history was obtained from the parental anamnesis recorded on the observation sheet, and congenital absence of teeth and supernumerary teeth were verified on the panoramic radiograph.

After applying these criteria, the final study group comprised 255 patients (136 girls, 119 boys), all presenting for the first time to an orthodontic service.

### 2.2. Clinical Examination and Evaluated Variables

Clinical examinations were performed using standard dental instruments. All clinical assessments, as well as the measurements performed on the study models, were carried out by two examiners (E.A.C. and R.T.M.), both specialists in orthodontics, with 23 and 3 years of clinical experience, respectively. No formal calibration procedure or assessment of inter-examiner agreement was performed. An observation sheet was completed for each patient, and the initial orthodontic documentation (panoramic radiograph, study models obtained from alginate impressions of both dental arches) was analyzed. Dental conditions affecting all teeth present on the arch were recorded, as were single-tooth changes and changes in dental occlusion. Tooth loss was classified as premature when a primary tooth was extracted at least one year before the eruption of the corresponding successor permanent tooth.

The following variables were evaluated for each patient: integrity of the occlusal support area; presence of dental caries on the primary teeth in the support area; premature extractions of primary teeth; the combination of caries and premature extractions; location of the damage (maxillary vs. mandibular arch); and the type of extracted primary molars (first, second, or both).

The support area was considered damaged when at least one of the following conditions was present: (a) caries on one of the primary teeth in the area, (b) premature loss of a primary tooth in the area, or (c) a combination of the two.

### 2.3. Statistical Analysis

Data were analyzed using IBM SPSS Statistics 25 (IBM Corp., Armonk, NY, USA), and graphical representations were generated using Microsoft Office Excel/Word 2024. Qualitative variables were expressed as case numbers and percentages. Comparisons between groups (by age category: 6, 7, 8, and 9 years, and by sex, respectively) were performed using Fisher’s Exact Test. Z-tests with Bonferroni correction were used to further detail the differences identified in the contingency tables. The threshold for statistical significance was set at α = 0.05.

Because no a priori sample size calculation was performed (see Section 2.1), post hoc calculations were used to document the precision and sensitivity of the analysis. The final sample (n = 255) allowed the overall prevalence of support area damage (72.2%) to be estimated with a 95% confidence interval (Wilson method) of 66.4–77.3%. With α = 0.05 and a statistical power of 80%, the sample could detect small-to-medium effects (Cohen’s w ≈ 0.18 for comparisons between sexes and w ≈ 0.21 for comparisons across the four age groups), whereas comparisons within smaller subgroups had lower power.

## 3. Results

Data from Table 1 present the characteristics of the analyzed patients. Most patients were 8 years old (41.2%) or 7 years old (25.9%) and were girls (53.3%). The occlusal support area was damaged in 72.2% of patients, most frequently through a combination of premature extractions and dental caries (52.7%). Among patients who presented with premature extractions only (31.5%), most cases involved extractions in the lower arch (60.3%). Among patients who presented with dental caries only (15.8%), the distribution by location was nearly equal, with a slightly higher number of cases in the upper arch (51.7%). Extracted primary molars were present in 19.6% of patients, with the second primary molar being the most frequently extracted (62%).

Data from Table 2 show the distribution of patients according to age and other analyzed parameters. Significant differences were observed between age groups for the following, according to Fisher’s Exact Tests with Bonferroni-corrected Z-tests:

- Support area damage (*p* = 0.032)—Patients with damage were significantly more frequently 8 years old than 6 years old (80% vs. 54.8%);
- Type of damage (*p* = 0.047)—Patients with premature extractions were significantly more frequently 6 or 7 years old than 9 years old (47.1%/40.4% vs. 11.1%);
- Extracted teeth (*p* = 0.009)—Patients with extracted teeth were significantly more frequently 7 years old than 9 years old (28.8% vs. 5.7%);
- Absence of damage (*p* = 0.032)—Patients without damage were significantly more frequently 6 years old than 8 years old (45.2% vs. 20%).

**Table 2:** Distribution of the patients according to age and other analyzed parameters.

Parameter	Age Group				*p* *
	6 Years	7 Years	8 Years	9 Years	
Support area					
Without damage	14 (45.2%)	19 (28.8%)	21 (20.0%)	17 (32.1%)	**0.032**
With damage	17 (54.8%)	47 (71.2%)	84 (80.0%)	36 (67.9%)	
**Type of damage**					
Premature extractions	8 (47.1%)	19 (40.4%)	27 (32.1%)	4 (11.1%)	**0.047**
Dental caries	3 (17.6%)	5 (10.6%)	13 (15.5%)	8 (22.2%)	
Both	6 (35.3%)	23 (48.9%)	44 (52.4%)	24 (66.7%)	
**Premature extractions**					
Upper arch	3 (37.5%)	6 (31.3%)	13 (48.1%)	1 (25%)	0.656
Lower arch	5 (62.5%)	13 (68.4%)	14 (51.9%)	3 (75%)	
**Dental caries**					
Upper arch	2 (66.7%)	4 (80%)	7 (53.8%)	2 (25%)	0.290
Lower arch	1 (33.3%)	1 (20%)	6 (46.2%)	6 (75%)	
**Extracted teeth**					
Absent	24 (77.4%)	47 (71.2%)	84 (80%)	50 (94.3%)	**0.009**
Present	7 (22.6%)	19 (28.8%)	21 (20%)	3 (5.7%)	
**Extracted teeth—type**					
1st primary molar	0 (0%)	2 (10.5%)	2 (9.5%)	1 (33.3%)	0.832
2nd primary molar	5 (71.4%)	11 (57.9%)	13 (61.9%)	2 (66.7%)	
Both	2 (28.6%)	6 (31.6%)	6 (28.6%)	0 (0%)	

* Fisher’s Exact Test; statistical significance was set at *p* < 0.05; statistically significant *p*-values are shown in bold.

Data from Table 3 show the distribution of patients according to sex and other analyzed parameters. None of the tested associations proved to be statistically significant (*p* > 0.05). Only a trend toward statistical significance was observed for the type of support area damage (*p* = 0.080), where, according to Bonferroni-corrected Z-tests, patients with premature extractions were more likely to be girls than boys (38.9% vs. 23.6%).

Data from Table 4 show the distribution of patients according to sex, within each age group. At 6 years, none of the tested associations proved to be statistically significant (*p* > 0.05). At 7 years, significant differences between sexes were observed for the type of damage (*p* = 0.001)—patients with premature extractions were significantly more frequently girls than boys (70% vs. 18.5%) while patients with both conditions (premature extractions and dental caries) were significantly more frequently boys than girls (66.7% vs. 25%)—and extracted teeth (*p* = 0.028)—patients with extracted teeth were significantly more frequently girls than boys (43.3% vs. 16.7%). At 8 years and at 9 years, none of the tested associations proved to be statistically significant (*p* > 0.05). These age-specific comparisons are based on small numbers of cases in several strata (e.g., 14 girls and 5 boys with isolated premature extractions at 7 years) and should be interpreted cautiously.

## 4. Discussion

The results of this study show a high prevalence of occlusal support area damage among children in early mixed dentition attending an orthodontic practice in Bihor County, Romania, most frequently through a combination of dental caries and premature extractions, with a significant increase in damage with age. Since the sample was recruited from a single orthodontic practice, these figures describe the clinical sample studied and should not be interpreted as population-based prevalence estimates for Bihor County. This high prevalence is consistent with the particular vulnerability of this area during the early mixed dentition period, consistently highlighted in the international literature [18,19]. At the same time, these findings fit within the broader context of pediatric oral health in Romania, where national and regional studies have consistently reported a high prevalence of dental caries in children aged 6–8 years, with caries experience of 84.3% in a survey of five major cities [20] and 77.7% in Mureș County [21], and have linked it to individual and socio-economic factors [17,22]. Comparable data from Western Romania point in the same direction, with the highest burden of active carious lesions observed in the 5–7-year age group [23].

The second primary molar was the most frequently extracted tooth, a finding consistent with data from the literature indicating this tooth as the most vulnerable to premature loss and the one with the most significant impact on dental arch dimensions [11]. A study dedicated exclusively to premature loss of the second primary molar showed that most of the associated space loss occurs within the first 12 months after extraction, underscoring the importance of close monitoring and early intervention during this period [24]. This aspect highlights the clinical importance of close monitoring and early intervention in cases of second primary molar extraction, particularly given the increased prevalence of this type of loss in our study group. The use of space maintainers represents the standard therapeutic approach recommended in these situations. A recent meta-analysis, which included 479 patients from 11 studies, showed that these devices effectively prevent migration of adjacent teeth and contribute to adequate masticatory function and speech development [25]. However, the available evidence remains relatively limited in scope, and the long-term effectiveness of different types of space maintainers continues to be debated in the literature [13].

An analysis of the distribution by age group reveals a steady increase in support area damage, from 54.8% at 6 years to 80% at 8 years, suggesting a cumulative effect of exposure to caries-related risk factors throughout the first phase of mixed dentition, consistent with data from the literature showing an approximately 30% increase in caries risk for each additional year of age [26]. A similar pattern of decline toward the end of the studied age range was also reported in a recent Romanian study conducted on children from Mureș County, where the prevalence of caries experience decreased from 77.7% in the 6–8-year age group to 48.2% in the 9–11-year age group [21]. At the same time, the proportion of isolated premature extractions decreased significantly with age, in favor of cases with combined damage (caries and extractions), while the proportion of extracted teeth without an erupted successor reached a maximum at 7 years and subsequently decreased at 9 years. This was probably because, toward the end of the studied interval, some of the teeth prematurely extracted at younger ages had already been replaced by their permanent successors [27]. This pattern underscores the importance of cautious interpretation of cross-sectional data, which capture both recent events and the outcome of biological processes that occurred prior to the examination, as the simultaneous measurement of exposure and outcome limits the possibility of causal inference [28].

The distribution by sex did not reveal statistically significant differences across the entire study sample, with the exception of a trend toward significance regarding the type of damage, which became statistically significant at the age of 7 years, with girls more frequently presenting isolated premature extractions and boys more frequently presenting combined damage. Data from the literature regarding the influence of sex on premature loss of primary teeth remain contradictory. Thus, Hernández-Palacios et al. reported a significantly higher risk in boys [29], while Murshid et al. did not identify significant differences between sexes [30], suggesting that individual and behavioral factors may have a greater influence than biological sex itself on the risk of premature primary tooth loss, consistent with the specialized literature indicating socio-economic status and behavioral habits as the dominant predictors of caries risk [31].

From a clinical perspective, the results of this study support the need for active monitoring of the occlusal support area starting at 6 years of age, given the rapid increase in the prevalence of damage up to 8 years. The early diagnosis of caries in the primary molars and prompt restorative treatment could prevent a significant proportion of premature extractions, particularly in the case of the second primary molar, the most frequently affected tooth in the study group. A German cohort study showed that, at 8 years of age, children enrolled in a continuous preventive dental care program had a prevalence of premature extractions of only 7.9%, compared with 41% in the control group [32]. In situations where extraction becomes unavoidable, the therapeutic decision regarding the use of a space maintainer should be individualized, taking into account the child’s age, the developmental stage of the successor tooth, and the time remaining until its eruption [13]. Last but not least, the trend observed among 7-year-old girls toward isolated premature extractions suggests that oral health education programs aimed at parents could benefit from an age-adapted approach, with particular emphasis on the 6–8-year period, identified in this study as critical for the development of the occlusal support area. Structured educational programs aimed at parents have already demonstrated effectiveness in improving knowledge and practices related to children’s oral health [33].

This study presents several limitations that should be noted. First, the cross-sectional design does not allow for the establishment of causal relationships between age, sex, and support area damage, but only the identification of associations. Second, the study group was recruited consecutively from a single specialized orthodontic practice in Oradea and not through population-based sampling. Children seeking orthodontic care may differ systematically from the general pediatric population in terms of occlusal characteristics, treatment needs, and access to dental care; for example, children with premature loss of primary molars or with malocclusion may be more likely to be referred for orthodontic evaluation, whereas children with limited access to care may be underrepresented. The direction and magnitude of the resulting selection bias cannot be determined from our data. Consequently, the findings should be interpreted as describing this clinical sample, and the prevalence estimates should not be extrapolated to the general pediatric population of Bihor County or Romania. Third, the clinical assessments and model measurements were performed by two examiners with different levels of experience, without prior calibration and without a formal assessment of inter- or intra-examiner reliability, which may have introduced measurement variability. Finally, the reduced size of some age and sex subgroups (for example, cases of premature extractions at 9 years) limited the statistical power and the stability of certain comparisons; the age-specific sex differences in particular should be regarded as exploratory, and non-significant associations should not be interpreted as a true absence of effect.

## 5. Conclusions

This study revealed a high prevalence of occlusal support area damage (72.2%) among children in the early mixed dentition attending an orthodontic practice in Oradea, Bihor County, Romania, most frequently through a combination of dental caries and premature extractions, with damage increasing significantly with age, from 54.8% at 6 years to 80% at 8 years, and the second primary molar being the most frequently prematurely extracted tooth. Sex-based differences were not statistically significant across the overall sample, except for an association at 7 years of age, where girls more frequently presented isolated premature extractions; this finding is based on small numbers and should be interpreted cautiously. These findings underscore the importance of active monitoring of the occlusal support area starting at 6 years of age and the need for early diagnosis and treatment of caries in the primary molars, as an essential measure for preventing premature extractions and their consequences on dental arch development, and may help inform preventive and interceptive programs tailored to the pediatric population of Bihor County, while population-based longitudinal studies with larger, multi-source samples are needed to confirm and extend these findings.

## Figures and Tables

**Table 1:** Characteristics of the analyzed patients.

Parameter (Nr., %)	Value
**Age**	
6 years	31 (12.2%)
7 years	66 (25.9%)
8 years	105 (41.2%)
9 years	53 (20.8%)
**Sex**	
Male	119 (46.7%)
Female	136 (53.3%)
**Occlusal support area**	
Damaged	184 (72.2%)
Undamaged	71 (27.8%)
**Type of damage**	
Premature extractions	58 (31.5%)
Dental caries	29 (15.8%)
Premature extractions & dental caries	97 (52.7%)
**Location—Premature extractions**	
Upper arch	23 (39.7%)
Lower arch	35 (60.3%)
**Location—Dental caries**	
Upper arch	15 (51.7%)
Lower arch	14 (48.3%)
**Extracted primary molars**	50 (19.6%)
**Type of extracted primary molars**	
1st primary molar	5 (10%)
2nd primary molar	31 (62%)
1st & 2nd primary molars	14 (28%)

**Table 3:** Distribution of the patients according to sex and other analyzed parameters.

Parameter	Sex		*p* *
	Female	Male	
Support area			
Without damage	41 (30.1%)	30 (25.2%)	0.574
With damage	95 (69.9%)	89 (74.8%)	
**Type of damage**			
Premature extractions	37 (38.9%)	21 (23.6%)	0.080
Dental caries	13 (13.7%)	16 (18.0%)	
Both	45 (47.4%)	52 (58.4%)	
**Premature extractions**			
Upper arch	16 (43.2%)	7 (33.3%)	0.579
Lower arch	21 (56.8%)	14 (66.7%)	
**Dental caries**			
Upper arch	5 (38.5%)	10 (62.5%)	0.272
Lower arch	8 (61.5%)	6 (37.5%)	
**Extracted teeth**			
Absent	110 (80.9%)	95 (79.8%)	0.875
Present	26 (19.1%)	24 (20.2%)	
**Extracted teeth—type**			
1st primary molar	3 (11.5%)	2 (8.3%)	0.840
2nd primary molar	15 (57.7%)	16 (66.7%)	
Both	8 (30.8%)	6 (25.0%)	
**Intact teeth**			
Absent	95 (69.9%)	89 (74.8%)	0.574
Present	41 (30.1%)	30 (25.2%)	

* Fisher’s Exact Test; statistical significance was set at *p* < 0.05. No statistically significant differences were observed; the trend for type of damage (*p* = 0.080) did not reach significance.

**Table 4:** Distribution of patients according to age, stratified by sex.

	6 y		7 y		8 y		9 y	
	F	M	F	M	F	M	F	M
**Support area**								
Without damage	7 (50%)	7 (41.2%)	10 (33.3%)	9 (25%)	14 (23.7%)	7 (15.2%)	10 (30.3%)	7 (35%)
With damage	7 (50%)	10 (58.8%)	20 (66.7%)	27 (75%)	45 (76.3%)	39 (84.8%)	23 (69.7%)	13 (65%)
*p* *	0.725		0.587		0.457		0.556	
**Type of damage**								
Premature extractions	4 (57.1%)	4 (40%)	14 (70%)	5 (18.5%)	15 (33.3%)	12 (30.8%)	4 (17.4%)	0 (0%)
Dental caries	2 (28.6%)	1 (10%)	1 (5%)	4 (14.8%)	6 (13.3%)	7 (17.9%)	4 (17.4%)	4 (30.8%)
Both	1 (14.3%)	5 (50%)	5 (25%)	18 (66.7%)	24 (53.3%)	20 (51.3%)	15 (65.2%)	9 (69.2%)
*p* *	0.377		**0.001**		0.911		0.336	
**Premature extractions—location**								
Upper arch	2 (50%)	1 (25%)	5 (35.7%)	1 (20%)	8 (53.3%)	5 (41.7%)	1 (25%)	0 (0%)
Lower arch	2 (50%)	3 (75%)	9 (64.3%)	4 (80%)	7 (46.7%)	7 (58.3%)	3 (75%)	0 (0%)
*p* *	1.000		1.000		0.704		– †	
**Dental caries—location**								
Upper arch	1 (50%)	1 (100%)	0 (0%)	4 (100%)	4 (66.7%)	3 (42.9%)	0 (0%)	2 (50%)
Lower arch	1 (50%)	0 (0%)	1 (100%)	0 (0%)	2 (33.3%)	4 (57.1%)	4 (100%)	2 (50%)
*p* *	1.000		0.200		0.592		0.429	
**Extracted teeth**								
Absent	11 (78.6%)	13 (76.5%)	17 (56.7%)	30 (83.3%)	51 (86.4%)	33 (71.7%)	31 (93.9%)	19 (95%)
Present	3 (21.4%)	4 (23.5%)	13 (43.3%)	6 (16.7%)	8 (13.6%)	13 (28.3%)	2 (6.1%)	1 (5%)
*p* *	1.000		**0.028**		0.085		1.000	
**Extracted teeth—type**								
1st primary molar	0 (0%)	0 (0%)	2 (15.4%)	0 (0%)	0 (0%)	2 (15.4%)	1 (50%)	0 (0%)
2nd primary molar	2 (66.7%)	3 (75%)	7 (53.8%)	4 (66.7%)	5 (62.5%)	8 (61.5%)	1 (50%)	1 (100%)
Both	1 (33.3%)	1 (25%)	4 (30.8%)	2 (33.3%)	3 (37.5%)	3 (23.1%)	0 (0%)	0 (0%)
*p* *	1.000		0.840		0.670		1.000	

* Fisher’s Exact Test (gender comparison within each age group); statistical significance was set at *p* < 0.05; statistically significant *p*-values are shown in bold. † Not applicable—zero cases in male group at 9 years.

## Data Availability

The data presented in this study are available on request from the corresponding author. The data are not publicly available due to privacy and ethical restrictions, as the dataset contains information collected from parents regarding their children.
